# Increased incidence of Graves’ disease during the COVID-19 pandemic in children and adolescents in the United States

**DOI:** 10.3389/fendo.2024.1426672

**Published:** 2024-12-05

**Authors:** Naama Pollack-Schreiber, Joanna S. Fishbein, Benjamin Udoka Nwosu, Parissa Salemi

**Affiliations:** ^1^ Division of Pediatric Endocrinology, Cohen Children’s Medical Center, NorthwellHealth, New Hyde Park, NY, United States; ^2^ Biostatistics Unit, Office of Academic Affairs, Northwell Health, New Hyde Park, NY, United States

**Keywords:** Graves’ disease, hyperthyroidism, auto-immune thyroid disease, COVID-19, SARS-CoV-2

## Abstract

**Introduction:**

Reports in adults indicate that Severe Acute Respiratory Syndrome Corona Virus-2 (SARS-CoV-2) infection and vaccination trigger the expression of autoimmune disease such as Graves’ disease, but the incidence of new onset Graves’ disease and its temporal relationship to the peaks of COVID-19 cases in children are unclear.

**Methods:**

This is a retrospective study of children and adolescents with new-onset Graves’ disease diagnosed between September 2017 and August 2022, N=156, mean age of 12.5 ± 4 year (y), with a range of 2.9-17.9y. There were 119 female (76.3%) and 37 male (23.7%) subjects. Subjects were categorized into 2 groups: pre-COVID-19 era Graves’ disease (n=63, age 12.5 ± 3.3y), and COVID-19 era Graves’ disease (n=93, age 12.4 ± 4.4y). We calculated incidence rate based on new cases of Graves’ disease and total number of new patient referrals to our endocrine clinic. We first compared the demographic, clinical and biochemical data between the above 2 groups; and also, between subjects with either a history of COVID-19 infection (n=23) or vaccination (n=17) to a control group (n=63).

**Results:**

The incidence of Graves’ disease was significantly higher during the pandemic: pre-COVID-19 versus the COVID-19 era, n=55, 0.56% vs n=93, 0.9%, p=0.005, after accounting for the total number of annual new patient referrals during the study period. The rise in the cases of Graves’ disease followed the spikes in the number of cases of COVID-19 in NY. There was also a statistically significant difference in the race distribution between the pre-COVID-19 and the COVID-19 era (p=0.026).

**Discussion:**

The incidence of Graves’ disease increased significantly in children living in New York during the COVID-19 pandemic. The temporal relationship between the peaks of COVID-19 cases and the increased cases of new onset Graves’ disease suggest possible autoimmune triggering by SARS-CoV-2.

## Introduction

Autoimmune disorders are characterized by the presence of autoantibodies and inflammatory reactions from the loss of immune tolerance and subsequent dysregulation of the immune system, leading to target organ damage and dysfunction (1). Graves’ disease results from a complex interaction between genetic predisposition, environmental factors, and the immune system. Thyroid stimulating hormone receptor antibody (TRAb) and/or thyroid stimulating immunoglobulin (TSI) stimulate the thyroid gland to produce excess thyroid hormone in Graves’ disease (2). Severe Acute Respiratory Syndrome Corona Virus-2 (SARS-CoV-2) infection and vaccination have been associated with the triggering of autoimmune diseases (1, 3, 4), particularly thyroid diseases (5, 6) such as Graves’ disease, following SARS-CoV-2 infection (7–9) or vaccination (10–14). These reports are mostly in adult patients, with very few publications in children (15, 16). Graves’ disease is rare in children and accounts for about 10% to 15% of cases of thyroid disorders in childhood. While there have been increasing reports of autoimmune thyroid disease following COVID-19 infection or vaccination, there is a dearth of knowledge on the incidence of new onset Graves’ disease and its temporal relationship to the peaks of COVID-19 cases in children during the pandemic.

The aim of this study was to determine possible changes in the incidence of Graves’ disease in children and adolescents living in New York before and during the COVID-19 pandemic era, and any association with the peaks of COVID-19 cases.

## Materials and methods

### Ethical approval

This study’s protocol was approved by the Feinstein Institute for Medical Research IRB (Docket #: 22-0728).

### Study design and subjects

Subjects were included in the study if they were <18 years of age and had a new diagnosis of Graves’ disease at the Division of Pediatric Endocrinology of the Cohen Children’s Medical Center, Northwell Health, between September 2017 and August 2022. The diagnosis of Graves’ disease was made based on features of autoimmune hyperthyroidism as marked by a suppressed thyroid stimulating hormone (TSH), elevated total thyroxine (TT4) or free thyroxine (free T4) level, and/or total triiodothyronine (TT3) concentration with a positive TSI and/or TRAb. Data was collected on subjects’ sex, race, date of diagnosis, age at diagnosis, individual and family history of either autoimmune diseases and/or thyroid disease, presenting signs and symptoms and the duration of the symptoms. Further biochemical and laboratory data included TSH, T4, free T4, TT3, anti-thyroid peroxidase antibody (TPOAb), anti-Thyroglobulin antibody (TGAb), TSI and TSH receptor antibodies, liver enzymes. We collected data on treatment with atenolol and methimazole, along with the duration and doses of each therapy. For subjects diagnosed in the COVID-19 era (starting from March 2020) we also collected information on COVID-19 infection and vaccine status in the year prior to their clinical presentation.

### Statistical analysis

Descriptive statistics were performed (e.g., frequency and proportion for categorical variables and means and standard deviations or median and interquartile range for continuous variables). We compared the demographic, clinical, and biochemical parameters of subjects between the groups, i.e., those diagnosed before COVID-19 pandemic (September 2017- February 2020) versus during the pandemic (March 2020-August 2022). We also compared subjects with a history of COVID-19 infection or COVID -19 vaccination in the year prior to their diagnosis with Graves’ disease to a control group, i.e., subjects diagnosed before the COVID-19 pandemic. A chi-square or Fisher’s exact test was used for categorical variables, and either a two-sample t-test or Wilcoxon rank sum test for continuous variables. The incidence rate was calculated as the number of new cases of Graves’ disease that presented to the Division of Pediatric Endocrinology divided by the total number of new-patient referrals made to the division. We computed the 95% exact binomial confidence interval and set the level of significance at p<0.05. All analyses were conducted using SAS software Version 9.4 (Cary, NC).

We reviewed the overall number of individuals that our division served for consultations, which includes Nassau and Queens Counties in New York, between 2018 and 2022. Population-level statistics for the 2 counties during the same period, specifically for the 5-19 year old sub-population, were equally reviewed and compared from census data (17, 18). The overall population decreased from 3.8 to 3.6 million individuals (17). Review of the percentile of pediatric population (5-19 years old) showed a similar proportion before and after the pandemic, with 15.8% in 2018 to 16% in 2022 in Queens County and 18.5% in 2018 to 18.4% in 2022 in Nassau County (18).We compared the number of new patients entering the clinic out of the total population of interest during that study period (by year) for Nassau and Queens Counties and found that in 2020 our Division of Endocrinology had a decline in the proportion of new patients seen as a percentage of the pediatric population in Nassau and Queens counties, when compared to each of the other years: 0.59% in 2020 vs 0.7% in 2018, 0.78% in 2019, 0.72% in 2021 and 0.76% in 2022. Therefore, we focused our calculation of incidence of Graves’ disease in the pediatric population on the subset of all new patients who presented to our division. Incidence for 2017 was not able to be computed as census data on population are reported by year, not month, and we only had 4 months of data for 2017.

## Results

One hundred and fifty-six (156) subjects were diagnosed with new-onset Graves’ disease between September 2017 and August 2022. The subjects’ demographic characteristics are described in **Table 1**, their clinical presentation profiles are summarized in **Table 2**, and their biochemical presentation summarized in **Table 3**. In order to analyze the thyroid function tests (TFT’s), we categorized each variable based on the free T4, Total T3 and Total T4 levels. If there was a discrepancy between the categorization, we prioritized the free T4 value, followed by the Total T3 and then Total T4 levels (**Table 4** **).** Of the 156 subjects diagnosed with Graves’ disease, 63 (40%) were diagnosed in the 30 months prior to the start of the pandemic (i.e., September 2017- February 2020), and 93 (60%) were diagnosed in the subsequent 30 months (March 2020-August 2022) during the pandemic era. The monthly rate of increase in Graves’ disease cases rose from 2 to 2.5 cases/month in the pre-COVID-19 era to 3.3 to 3.5 cases/month in the COVID-19 era. The incidence of Graves’ disease in this cohort in the COVID-19 era was significantly higher than the incidence in the pre-COVID-19 era, 0.90% vs 0.56%, p=0.005. (**Table 5** **).**

**Table 1:** Baseline demographic characteristics of the subjects stratified by pre-COVID-19 era and COVID-19 era.

Variables		Total	Pre-COVID-19 era	COVID-19 era	p-value
		156	63	93	
Gender *	**Female**	119 (76.3%)	51 (81%)	68 (73.1%)	0.34
	**Male**	37 (23.7%)	12 (19%)	25 (26.9%)	
Ethnicity *	**White**	53 (33.9%)	18 (28.6%)	35 (37.6%)	0.026
	**Asian**	49 (31.4%)	17 (27%)	32 (34.4%)	
	**African American**	18 (8.3%)	9 (14.3%)	9 (9.7%)	
	**Hispanic/Latino**	27(17.3%)	11(17.4%)	16(17.2%)	
	**Unknown/more than one race**	9 (5.7%)	8 (12.7%)	1(1.1%)	
Age at presentation **		12.5 (4.0)	12.5 (3.3)	12.4(4.4)	0.48
Personal history of autoimmune disease *		8 (5.1%)	1 (1.6%)	7 (7.5%)	0.14
Family history of autoimmune disease *		87 (55.8%)	35 (55.6%)	52 (55.9%)	1
Family history of thyroid disease *		81 (51.9%)	35 (55.6%)	46 (49.5%)	0.51
Family history of thyroid disease in 1st degree relative *		53 (34%)	26 (41.3%)	27 (29%)	0.12

*Number of subjects, percentiles; **Mean, standard deviation.

**Table 2:** Comparative analysis of the subjects’ presenting symptoms and signs stratified by pre-COVID-19 era and COVID-19 era.

Variables		Total	Pre-COVID-19 era	COVID-19 era	p-value
		156	63	93	
**Symptom** duration *	**0-3 months**	71 (45.5%)	24 (38.1%)	47 (50.5%)	0.48
	**4+ months**	26 (16.7%)	12 (19%)	14 (15.1%)	
	**Asymptomatic**	14 (9%)	6 (9.5%)	8 (8.6%)	
	**Unknown**	45 (28.8%)	21 (33.3%)	24 (25.8%)	
Presenting symptoms *	**Palpitations**	48 (30.8%)	15 (23.8%)	33 (35.5%)	0.15
	**Shortness of breath/chest tightness/pain**	13 (8.3%)	7 (11.1%)	6 (6.5%)	0.38
	**Heat intolerance/Sweating**	59 (37.8%)	21 (33.3%)	38 (40.9%)	0.4
	**Weight loss**	75 (48.1%)	31 (49.2%)	44 (47.3%)	0.87
	**Increased appetite**	31 (19.9%)	12 (19%)	19 (20.4%)	1
	**Fatigue/weakness**	30 (19.2%)	16 (25.4%)	14 (15.1%)	0.15
	**Emotional lability/anxiety**	31 (19.9%)	16 (25.4%)	15 (16.1%)	0.16
	**Concentration problems/** **change in school performance/hyperactivity**	38 (24.4%)	13 (20.6%)	25 (26.9%)	0.45
	**Sleep problems**	14 (9%)	8 (12.7%)	6 (6.5%)	0.25
	**Diarrhea**	23 (14.7%)	10 (15.9%)	13 (14%)	0.8
Presenting signs *	**Exophthalmos**	22 (14.1%)	9 (14.3%)	13 (14%)	1
	**Hand tremor**	40 (25.6%)	11 (17.5%)	29 (31.2%)	0.06
	**Goiter**	122 (78.2%)	52 (82.5%)	70 (75.3%)	0.32
	**Tachycardia**	105 (69.1%)	41 (65.1%)	64 (71.9%)	0.38

*Number of subjects, percentiles.

**Table 3:** Comparative analysis of the subjects’ biochemical presentation stratified by pre-COVID-19 era and COVID-19 era.

Variables		Total	Pre-COVID	Post-COVID	p-value
**Thyroid hormone abnormality** by severity*	Normal	3 (1.9%)	0 (0%)	3 (3.2%)	0.48
	Mild	14 (9%)	5 (7.9%)	9 (9.7%)	
	Moderate	52 (33.3%)	24 (38.1%)	28 (30.1%)	
	Severe	87 (55.8%)	34 (54%)	53 (57%)	
Anti TPO (IU/mL)*	Negative (<35)	22 (17.6%)	10 (20.4%)	12 (15.8%)	0.4
	35-100	16 (12.8%)	4 (8.2%)	12 (15.8%)	
	101-500	34 (27.2%)	17 (34.7%)	17 (22.3%)	
	501-1000	19 (15.2%)	7 (14.3%)	12 (15.8%)	
	>1000	34 (27.2%)	11 (22.4%)	23 (30.3%)	
Anti-thyroglobulin (IU/mL)*	Negative (<40)	45 (38.5%)	14 (31.8%)	31 (42.5%)	0.65
	41-100	16 (13.7%)	7 (15.9%)	9 (12.3%)	
	101-500	34 (29.1%)	14 (31.8%)	20 (27.4%)	
	501-1000	5 (4.3%)	3 (6.8%)	2 (2.7%)	
	>1000	17 (14.5%)	6 (13.6%)	11 (15.1%)	
Thyroid stimulating immunoglobulin (IU/L)**		9.3 (4.1-21.4)	9.4 (3.8-24)	9.3 (4.2-19.3)	0.87
TSH receptor antibody (IU/L)**		12.2 (6.1-22)	15.8 (6.7-23.2)	11.3 (6.1-21.3)	0.62

*Number of subjects, percentiles; **Median, interquartile range.

**Table 4:** Thyroid function tests categorization.

	Free T4 (ng/dL)	Total T3 (ng/dL)	Total T4 (ug/dL)
**Normal**	<1.9	<200	<12
**Mild**	1.9 to <2.5	200 - 300	12-14
**Moderate**	2.5 to <4.4	>300 to 400	>14-18
**Severe**	>=4.4	>400	>18

**Table 5:** Incidence of new onset Graves’ disease cases in the pre-COVID-19 era versus the COVID-19 era.

Time	Total number of new patient referrals to the Pediatric Endocrinology Division	Total number of subjects with new-onset Graves’ disease	p-value
**Pre-COVID-19 era** 1/1/2018- 2/29/2020	9830	55 (0.56%)	0.005
**COVID-19 era** 3/1/2020 - 8/31/2022	10375	93 (0.90%)	
**Total**	20205	148	

More White and Asian subjects were diagnosed with new-onset Graves’ disease than African American subjects during the pandemic, (p=0.026), (**Table 1**). Additionally, more subjects presented with fatigue/weakness (p=0.15) and emotional lability/anxiety (p=0.16) in the pre-COVID-19 era, while more individuals presented with palpitations (p=0.15) and tremor (p=0.06) in the COVID-19 era (**Table 2**).

### Positive Versus Negative History of COVID-19 Infection/vaccination

When evaluating for a prior COVID-19 infection/vaccination, 23/93 (24.7%) subjects reported a previous COVID-19 infection, and 17/93 (18.2%) subjects reported COVID-19 vaccination, in the year prior to diagnosis. Out of these subjects, 5 reported both. 63 subjects were diagnosed with Graves’ disease before the pandemic and served as the control group.

Some differences in the clinical presentation were noted between the groups as shown in **Table 6**. In the group with a reported prior COVID-19 infection, a higher percentage of subjects reported concentration problems, irritability or changes in school performance (p=0.03). Similar increases were seen in the vaccinated group. Those vaccinated had a higher percentage of exophthalmos at presentation, and a lower percentage of goiter.

**Table 6:** Comparative analysis of the subjects’ presenting symptoms and signs between subjects with prior infection/vaccination status and the controls.

Variables		Control group	Previous COVID-19 infection	p-value	Previous COVID-19 vaccination	p-value
		63	23		17	
Presenting symptoms *	**Palpitations**	15 (23.8%)	6 (26.1%)	1	6 (35.3%)	0.36
	**Shortness of breath/chest tightness/pain**	7 (11.1%)	3 (13%)	1	0 (0%)	0.33
	**Heat intolerance/Sweating**	21 (33.3%)	8 (34.8%)	1	6 (35.3%)	1
	**Weight loss**	31 (49.2%)	13 (56.5%)	0.63	10 (58.8%)	0.59
	**Increased appetite**	12 (19.1%)	7 (30.4%)	0.38	4 (23.5%)	0.74
	**Fatigue/weakness**	16 (25.4%)	4 (17.4%)	0.57	4 (23.5%)	1
	**Emotional lability/anxiety**	16 (25.4%)	2 (8.7%)	0.13	3 (17.7%)	0.75
	**Concentration problems/** **change in school performance/hyperactivity**	13 (20.6%)	11 (47.8%)	0.03	7 (41.2%)	0.11
	**Sleep problems**	8 (12.7%)	1 (4.3%)	0.43	0 (0%)	0.19
	**Diarrhea**	10 (15.9%)	3 (13%)	1	2 (11.76%)	1
Presenting signs *	**Exophthalmos**	9 (14.3%)	3 (13%)	1	5 (29.4%)	0.16
	**Hand tremor**	11 (17.5%)	6 (26.1%)	0.37	3 (17.7%)	1
	**Goiter**	52 (82.5%)	17 (73.9%)	0.37	10 (58.8%)	0.052
	**Tachycardia**	41 (65.1%)	15 (68.2%)	1	13 (81.2%)	0.25

*Number of subjects, percentiles.

## Discussion

This study found a statistically significant increase in the incidence of new-onset Graves’ disease between the pre-COVID-19 and COVID-19 era (p=0.005), when compared with the total number of new patient referrals to the pediatric endocrine clinic. This is the largest study investigating the incidence of new-onset Graves’ disease during the COVID-19 pandemic in the pediatric population, as well as the longest in duration in both the pediatric and adult population.

The mechanism of this increased incidence in Graves’ disease during the COVID-19 era is unclear. However, several mechanisms have been proposed to lead to autoimmunity following a pathogenic virus infection or vaccination in different mechanisms, mainly molecular mimicry (1, 3, 19–21). A systematic review also suggests that cytokine storm, and the expression of angiotensin-converting enzyme 2 (ACE2) may play a significant role in the pathogenesis of Graves’ disease (22).

SARS-CoV-2 is an enveloped virus that can invade the cell by attaching to the host cell receptor, ACE2 (1). A recent study investigating expression analysis of ACE2 in 31 tissues derived from healthy individuals found that ACE2 protein expression is widely detected in various types of tissues and upregulated in thyrocytes (5). In a different study, ACE-2 messenger ribonucleic acid (mRNA) was detected in all surgical samples of 15 thyroid glands of patients with nodular goiter (23). It is plausible that the thyroid gland is affected by SARS-CoV-2 and can lead to thyroid dysfunction. Therefore, it is possible that the increased incidence found in our study during COVID-19 era was due to the triggering of the expression of autoimmune thyroid disease by exposure to SARS-CoV-2.

**Figure 1:**
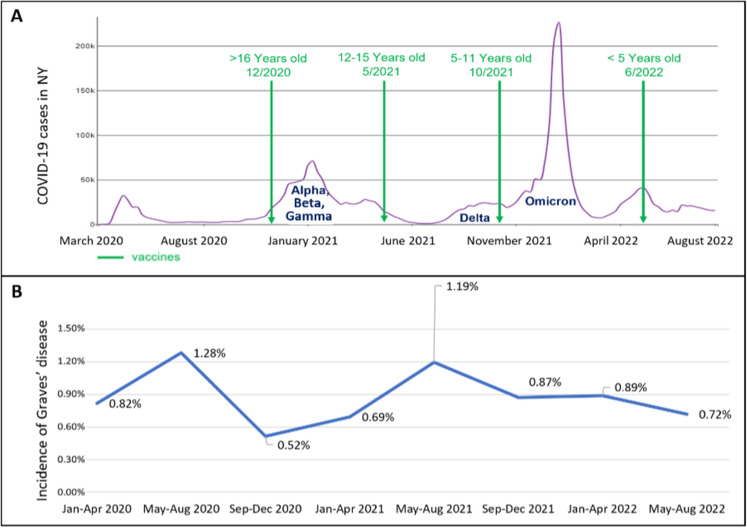
**(A, B)** The timeline of the COVID-19 era. **(A)** shows the number of COVID-19 cases diagnosed overtime and the SARS-COV-2 variants in NY as extracted from the CDC database (24), and the dates vaccinations were approved for use in children and adolescents. **(B)** shows the incidence of new onset Graves’ disease cases in the pediatric endocrinology division. There is a temporal relationship between the spikes in COVID-19 cases and subsequent increase in incidence of cases of new-onset Graves’ disease.

Additionally, vaccines work by triggering the immune response, which may stimulate a hyperinflammatory condition (4). The COVID-19 vaccines approved for children and adolescents >6 months of age in the United States are mRNA vaccines consisting of the genetic code for the spike protein antigen. Their approvals were gradual, as shown in **Figure 1** (24). There had been reports of cases of new-onset Graves’ disease following COVID-19 infection and or vaccination (7–14, 25).

A matched case control study done in adult population did not find an association between COVID-19 vaccination and the incidence of Graves’ disease (26). One publication reported an increased incidence of Graves’ disease in 2021 vs 2017-2019 in adults (27), and another smaller pediatric study reported an increased incidence as well as more patients with palpitations and tremor (28). Our data is similar to the above clinical finding of increased palpitations (p=0.15) and tremor (p=0.06) during the pandemic.

We found that an increase in the number and incidence of new-onset Graves’ disease cases occurred a few months after the peak of COVID-19 cases, specifically after the first peak in March-April 2020 and after the peak in January 2021 that included the Alpha, Beta and Gamma variants as detailed in **Figure 1**. This again suggests the possibility that SARS-CoV-2 could trigger autoimmune thyroid disease, especially Graves’ disease. We further examined the incidence rates in the years preceding the COVID-19 era to provide robust comparative data for the COVID-19 era incidence. There was no additional peak in new Graves’ disease cases after the peak in COVID-19 cases in January 2022 due to the Omicron variant which may imply an altered immune response to new variants or a protective effect of vaccination. One study demonstrates that individuals who contracted SARS-CoV-2 in the early stages of the pandemic exhibited a higher prevalence of immunological abnormalities compared to those infected during later phases of the pandemic (29).

There was a statistically significant difference in the ethnicity/race distribution between the pre-COVID-19 era and the COVID-19 era (**Table 1**). The percentage of African American subjects with new onset Graves’ disease decreased during the pandemic, and the percentage of White and Asian subjects increased, despite the race distribution remaining stable in our catchment area (18). A possible explanation can be genetic polymorphisms in the ACE2 gene which is suggested as one of the factors that changes the receptor stability and vulnerability to the virus and the clinical presentation after COVID-19 infection (30–33).

Specifically in the African American population, reduced molecular expression of ACE2 has been reported (30), which might affect the virus’ invasion of the thyrocytes. This may suggest important clinical implications as to whether certain polymorphisms may have a protective effect against viral invasion as opposed to others. Further research is needed to evaluate for these racial differences due to small number of African American patients in the current study.

A statistically significant increase in concentration problems, irritability or change in school performance was seen in those with a prior COVID-19 infection (**Table 6**). A confounder might be the effect of the pandemic on school performance in general as described in a meta-analysis (34), but when comparing the pre-pandemic to the post-pandemic group, there was no such difference (**Table 2**). A possible explanation can be long-COVID, as one of the symptoms described is concentration problems (35).

Exophthalmos is the most common extrathyroidal manifestation of Graves’ disease (25%). Other extrathyroidal manifestations include thyroid dermopathy (1.5%) and acropachy (0.3%) (36). We found a higher percentage of subjects presenting with exophthalmos in those that were vaccinated (p=0.16), (**Table 6**). Review of the literature shows that multiple case reports were published reporting thyroid eye disease following COVID-19 vaccination (37–40). No patients in the current study reported other extrathyroidal manifestation.

One study (41) showed an increase of severity during the COVID-19 era in patients with new onset autoimmune hypothyroidism, mostly related to difficulties in access to healthcare services. We did not find differences in the severity of clinical or biochemical presentation between the pre COVID-19 and the COVID-19 era.

Our study has several limitations which include its retrospective design, which precludes causation. Since the population of patients who attend our endocrine clinic is not generalizable, the incidence estimates used in our study may not reflect true incidence in the population. However, the fact that the proportion of the general population who were seen as new patients in our endocrine clinic remained relatively stable from 2018 to 2022 (≈0.7 to 0.78%) supports the premise for an increased incidence of new cases of Graves’ disease in Queens and Nassau County in the COVID-19 era. Some confounders need to be taken into consideration. As discussed in the methods section, there was no significant population change (17, 18) or change in the healthcare seeking behavior to our practice (**Table 5**) that can explain the change in incidence. Another cofounder is physical and psychological stress, which is a risk factor in the pathogenesis of different autoimmune disorders and can trigger new onset or exacerbation of those diseases (42, 43). It is possible that the sedentary lifestyle and dietary change during the pandemic had an effect as well. Studies showed an increased incidence of different autoimmune diseases in patients less engaged in physical activity (44). The relationship between diet and autoimmunity was also investigated. The temporal relationship we found between the number of COVID-19 cases and the number of new onset Graves’ disease cases is less likely to be explained by those confounders (45, 46).

This study was performed on data from a specific geographic area. Even though New York’s population is relatively diverse as shown in the race/ethnicity distribution, this may limit the generalizability of the findings to other regions. Multicenter study across different populations can help validate the findings.

Further limitation includes the relatively small sample of patients with previous COVID-19 infection/vaccine. A prospective study comparing patients with new onset Graves’ disease after a SARS-CoV-2 infection or vaccination versus unexposed patients is now in progress by our group and could provide a better understanding of risk factors and presenting clinical characteristics.

The study’s strengths include it being the largest and longest retrospective pediatric study on the incidence of Graves’ disease in the pediatric population associated with the COVID-19 pandemic. Additionally, there is a comparison of the timing of the peaks in the number of subjects presenting with new onset Graves’ disease with the peaks of new cases of COVID-19 in New York state as reported by the CDC (24).

In conclusion, our study provides evidence that the incidence of Graves’ disease increased significantly in our institution during the COVID-19 era in children and adolescents in the US. The temporal relationship between increases in the number of COVID-19 cases and the elevated incidence of new onset Graves’ disease could suggest the possibility of triggering of autoimmune thyroid disease by SARS-CoV-2 virus. However, mechanistic studies are needed to provide conclusive evidence.

## Data Availability

The raw data supporting the conclusions of this article will be made available by the authors, without undue reservation.
